# Supplementary material for: Complexome profiling of the Chlamydomonas psb28 mutant reveals TEF5 as an early PSII assembly factor
Source: Plant Cell. 2025 Mar 17;37(6):koaf055. doi: 10.1093/plcell/koaf055 (PMC12164590; doi:10.1093/plcell/koaf055)
Supplement: koaf055_Supplementary_Data [file koaf055_supplementary_data.zip › psb28_interactive.zip!/plots/intensity/qualityPlots/peptides/AAI1_Cre10.g434750.t1.2.html]

### Cre10.g434750.t1.2

Trivial name: AAI1  
SeqCoverage [median]: 9.70  
Mass: 60.21 kDa  
  
 

| Sequence | MissedCleavage | Mass [Da] | wtIntensity | muIntensity | Score |
| --- | --- | --- | --- | --- | --- |
| DINVVLVAPK | 0 | 1066.64 | 577.69 | 1449.57 | 71.69 |
| DSIAEAGMDIK | 0 | 1148.54 | 0.00 | 1319.84 | 90.91 |
| FGPTEEYIVR | 0 | 1209.60 | 689.44 | 5757.27 | 109.71 |
| GMLSVYNSFNEADKK | 1 | 1701.80 | 994.82 | 3527.21 | 91.21 |
| SVVQAVQR | 0 | 885.50 | 1980.30 | 5876.50 | 110.63 |
|  |  |  | 4242.2 | 17930.4 |  |
